# Developing HIV assisted partner notification services tailored to Mexican key populations: a qualitative approach

**DOI:** 10.1186/s12889-021-10612-3

**Published:** 2021-03-20

**Authors:** Heleen Vermandere, Santiago Aguilera-Mijares, Liliane Martínez-Vargas, M. Arantxa Colchero, Sergio Bautista-Arredondo

**Affiliations:** Division of Health Economics and Health Systems Innovations, National Institute of Public Health, Avenida Universidad 655, Colonia Santa María Ahuacatitlán, 62100 Cuernavaca, Morelos Mexico

**Keywords:** Partner notification, HIV, Men who have sex with men, Transgender persons, Mexico

## Abstract

**Background:**

Assisted partner notification services (APNS) may increase HIV testing, early diagnosis, and treatment, but they are not formally implemented in Mexico, where the HIV epidemic is concentrated in men who have sex with men (MSM) and transwomen (TW). This study aimed to explore the awareness of and need for HIV partner notification, as well as to outline potential strategies for APNS based on identified barriers and facilitators.

**Methods:**

We conducted semi-structured interviews to explore partner notification with MSM, TW, and counselors. Afterwards, brainstorm sessions were carried out to produce strategies for implementing APNS.

**Results:**

Most participants reported experiences with informal partner notification and serostatus disclosure, but not with APNS. Only one counselor indicated assisting notification systematically. The main barriers for notifying or disclosing mentioned by both MSM and TW included fear of (violent) reactions, discrimination and lacking contact information of casual partners. Participants thought it was easier to inform a formal partner, conditional of being well informed about HIV. Given current stigma and lack of awareness, it was suggested that APNS should be preceded by HIV awareness efforts, and be provided by counselors or peers to mitigate potential rejection or violent reactions.

**Conclusions:**

While APNS are not formally implemented in Mexico, all participants supported the service, indicating that APNS could potentially enhance early HIV diagnosis in Mexico. Strategies to implement such services need to be flexible addressing the individual needs of participants, guaranteeing the safety of more vulnerable participants.

**Supplementary Information:**

The online version contains supplementary material available at 10.1186/s12889-021-10612-3.

## Background

Early diagnosis is key to treat HIV successfully [1]. It allows for early initiation of antiretroviral therapy (ART), which has been found to reduce the risk of HIV transmission [2], development of a non-AIDS-defining illness, progression to AIDS, or death [3]. Instead, the unawareness of HIV-positivity has been linked to high-risk sexual behaviors [4]. However, despite the expansion of HIV testing services over the last decades [5], diagnosis remains one of the most elusive targets worldwide [6] to reach the UNAIDS (Joint United Nations Program on HIV/AIDS) 90–90-90 goals for 2020 [7]. As of 2019, only 81% of worldwide people living with HIV (PLWH) knew their status, 82% of these were on ART, and 88% of these had viral suppression [8]—which is when HIV is sexually untransmittable [9]—as opposed to the 90% aimed for each indicator.

In order to undertake the challenge of scaling up HIV diagnosis, new approaches to improve the efficiency and coverage of HIV testing services are needed. This is can be difficult because PLWH do not always present symptoms that lead to seeking health services and they often belong to unsuccessfully reached key populations. HIV assisted partner notification services (APNS) have been strongly recommended by the World Health Organization as one such approach [10]. APNS are services offered to inform partners of a person diagnosed with HIV that they are at risk of having HIV and should seek testing [11].

A systematic review of randomized controlled trials concluded that APNS can increase HIV testing services uptake, which can result in a higher proportion of PLWH being diagnosed and linked to care; whereas reported social harm and adverse events due to the service have been rare [12]. Sixty-seven countries worldwide (six in Latin America) included HIV APNS in their national policies, as of 2016 [13]. Regarding key populations, APNS have been found to be highly accepted by men who have sex with men (MSM) and transwomen (TW) in Spain [14]; while MSM in Australia found APNS to be difficult, fearing potential repercussions, but they believed it is the right thing to do and required professional support to carry it out [11]. As for Latin America, Peruvian studies have reported that most MSM and TW considered it important to notify partners (more so formal than casual ones) [15]; and that reasons for failure to notify varied by partner type, including not understanding the importance of notification, embarrassment, fear of rejection, and inability to find the partner [16]. However, there remains an important gap in the literature about APNS in Latin American key populations.

While APNS seem to have the potential to increase HIV testing, early diagnosis, and treatment, they have yet to be formally applied in the context of concentrated epidemics in low- and middle-income countries such as Mexico. In this Latin American country, national government guidelines stipulate that users testing HIV+ should be informed about the importance of sharing their status with sexual partners, for them to be linked to health services [17]; and that it is recommended to provide APNS during medical follow-up visits of PLWH [18]. Yet despite this and some isolated efforts by civil society organizations to carry out APNS [19], this strategy has not been standardized and implemented systematically in all settings offering HIV testing.

In Mexico, by 2018 only 80% of PLWH knew their diagnosis [20]. To be effective, APNS would have to be tailored to the Mexican key populations—MSM and TW having the highest prevalence [21–24]. A nation-wide study in 2011 found only 32% of MSM living with HIV knew their diagnosis [22]; another study in Mexico City, in 2012, found the same was true for only 26% of TW living with HIV in sex partner meeting places [23]. As a result, the HIV epidemic in Mexico is driven by undiagnosed and untreated key populations who continue to unknowingly transmit the virus [25].

Both MSM and TW face the barriers of stigma and discrimination in Mexico, due to their sexual orientation and gender identity [26–28]. The same applies to PLWH [29], meaning that the need for confidentiality in APNS is of utmost importance, albeit services also need to be accommodated for differences in the underlying correlates of HIV risk and sexual health needs between MSM and TW [30].

The lack of systematically applied APNS is a missed opportunity in the public health response to HIV in Mexico, given that they could contribute to trace down potentially infected partners of recently diagnosed people. Trials have reported APNS resulted in higher proportions of identified partners diagnosed with HIV following testing [12]. This study therefore aimed to explore the awareness of and need for HIV partner notification among MSM and TW from the Metropolitan Area of Mexico City, as well as to outline potential strategies for APNS based on identified barriers and facilitators.

## Methods

### Data collection procedures

For this exploratory qualitative study, semi-structured interviews were conducted with MSM and TW, using a guide to explore core themes: their experience with partner notification of HIV diagnosis, the barriers and facilitators surrounding it, and their suggestions for APNS. In addition, interviews were carried out with counselors to explore their experience with APNS, as well as their suggestions on how to implement them. The interview guides, developed by our research team as part of the study, are included in Annex 1.

The interviews took 30–45 min and they were conducted between January and February 2019 at an AIDS Service Organization (ASO; a local non-governmental organization). They were carried out by a research assistant trained in qualitative methods and respectful of sexual diversity. Participation was voluntary, all participants provided a signed informed consent, and the study was approved by the Ethical Committee from the National Institute of Public Health of Mexico.

### Recruitment of participants

We conducted a criterion sampling [31] to purposefully include the participation of three profiles involved in HIV partner notification: PLWH, HIV-negative people, and counselors. The eligibility criteria for the first two profiles included being at least 18 years-old and self-identifying as a TW or a cisgender man reporting at least one sexual encounter with another cisgender man in the previous 12 months. The counselors were at least 18-years old and had at least one year of work experience in the HIV testing and counseling area.

First, the recruitment was carried out by the staff of an ASO inside two public HIV clinics in Mexico City. They screened for eligibility, explained the study, and scheduled an interview for those who expressed interest in participating. After this, a snowball sampling was also carried out, in which the staff used their personal networks to recruit participants, with the purpose of balancing the participation between the three chosen profiles. The interviews took place in the buildings of the ASO and were conducted by research assistants who were supervised by senior researchers.

### Data analysis

All interview audio-recordings were transcribed verbatim and uploaded into MAXQDA 11 Plus, a qualitative analysis software. Data underwent a conventional qualitative content analysis [32], which avoids preconceived categories and instead follows an inductive category development. First, the data was reviewed to identify emerging codes, with which an initial codebook was created that included definitions for each code. Next, two researchers carried out an intercoder agreement exercise to refine the codebook and coding procedure: 10% of interviews were coded, seeking an agreement equal to or above 80%. Once this was accomplished, we proceeded to code all data; and, afterward, each coded segment was synthesized in order to identify categories (i.e., the grouping of different codes into meaningful clusters based on how they relate).

The participant’s quotes presented in this paper were translated from Spanish using the back-translation method, in order to obtain an English version that is equivalent to the source in meaning and in tone. This method first requires that a bilingual person translates into the target language, then a second bilingual person translates the material back into the original language, and this process is iterated to check for quality and make adjustments in the translation [33].

### Strategies for implementing APNS

Having identified the key components related to APNS, research team members and project partners (ASOs’ staff and counselors) conducted brainstorming sessions in order to develop prototype strategies for the efficient implementation of APNS directed at MSM and TW in Mexico.

## Results

Between January and March 2019, a total of 28 interviews were conducted among 12 MSM, eleven TW, and five counselors, all of whom resided in the Mexico City Metropolitan Area. MSM had an average age of 30 years and half of them were living with HIV. TW were on average 43 years old and six of them were living with HIV. Three counselors worked in ASOs and two in public health services.

Other participant characteristics were identified throughout the interviews: some MSM were volunteers for ASOs, and some TW mentioned previous or current involvement with transactional sex, homelessness, substance abuse, or prison.

The following results present similarities and differences between MSM and TW; their quotes include the HIV status and identifier number of the participant. No differences were identified between participants living with HIV and HIV-negative participants. Quotes of counselors are indicated by “C” and their identifier number.

### Experiences

Since APNS were not an established service in Mexico at the time of the study, two kind of experiences emerged during the interviews among MSM and TW: 1) informal partner notification (i.e., discussion of a new HIV diagnosis with recent sex partners, not guided by a provider); and 2) serostatus disclosure (i.e., discussion of HIV status with a new sexual partner). Serostatus disclosure is deemed relevant for exploration and included in the analysis on account of the context of absent APNS and of its closeness to partner notification.

A total of nine MSM and three TW mentioned having experience with informal partner notification or serostatus disclosure (view Table 1). Four MSM reported having informed their partners, three had been informed, two had experienced both informing and being informed, and three had no experience. Three TW had informed a partner, one of them indirectly though, by prison staff notifying on her behalf. One TW had been informed, one had experience in both directions, and six reported no experience (although two of them mentioned notifying a partner of a syphilis infection). Only three MSM and one TW were advised to notify by health care providers; one of both the MSM and TW was told that notification was optional. *“They also told me that if I don’t want to say it [living with HIV] it’s my right; I just had to use a condom from now on”* (+TW17). Almost all participants living with HIV had informed at least one partner (*n* = 5).

**Table 1:** Participant’s experience with informal HIV partner notification or serostatus disclosure

	MSM	TW	Total
**HIV Status**			
HIV-	6	5	11
HIV+	6	6	12
**Notification experience**			
Notifying/disclosing	4	3^a^	7
Being notified/someone discloses to you	3	1	5
Both	2	1	4
None	3	6^b^	9

^a^One TW notified indirectly by asking prison staff to inform her partner

^b^Two TW reported having notified their syphilis diagnosis to their partners

Some MSM and TW said that the disclosure happened at the beginning of a relationship and face to face. *“I told him that, well, if the couple thing can work, let’s go ahead. So he mentioned that, well, he had an issue; and we arranged to meet, talked, and then he told me he was positive”* (−MSM6). In some cases, the partner who was disclosed was also living with HIV, which gave them confidence to disclose. *“He [partner] started telling me… that he took a medicine; and I said: ‘This is the moment, he has told me now [his positive status]... I feel more trust’, and I dared telling him [about her positive status]”* (+TW17).

Others indicated that notification occurred during hospital admissions due to HIV symptoms. *“I said to him [while admitted in a hospital]: ‘The thing is the nurse told me that I have HIV’; so at that moment we both started crying”* (+TW13). One TW had an experience of an intervention resembling APNS: she shared that officials at the detention center notified her partners through a “*program*”, with her authorization, after she was diagnosed; adding that her partners were subsequently tested as well.

As for the counselors’ experiences, some indicated they suggest newly HIV-diagnosed clients to notify their status to partners potentially at risk. However, only one had heard about APNS as a service and in his work it was standard practice to request consent for providers to invite clients’ partners to get tested, and to provide users with tools on how to notify themselves.

*Our informed consent has a section where they can place the name of their sex partners and their phone number, and where they authorize or not if we can contact them to invite them to get tested… The psychologist can give them [clients] tools to share the diagnosis with their partners… The counseling providers are also trained to provide elements to the client regarding ‘how to share my diagnosis, who to share it with, what to do’… underlining the importance of the diagnosis being personal, being confidential.* (C18)

Other providers stated that, in addition to recommending notification, on a few occasions they have offered or have been requested to support clients during the notification process. As such, providers have employed different notification strategies: a) contacting the partner to offer HIV testing, with or without mentioning the client; b) being present when the client notifies a partner; and c) providing counseling to the client and partner after the notification took place.

*On a few occasions, yes, I’ve had cases [requesting support for notification] … A [trans] girl that, well, her husband didn’t know… When she decided to take him to the clinic… she had told me: ‘I will take him to keep me company, but you will do your job’ [inviting him to get tested without revealing her diagnosis], right? So I had to speak to him.* (C14)

### Barriers and facilitators

#### Partner reactions

The fear of HIV stigma and people’s negative reactions stood out as the main barrier for notification or disclosure in MSM and TW. Most participants believed HIV stigma or “taboo” are still common and severe among Mexican society. *“Unfortunately, there’s people that are still living with the taboo; and the taboo I think is the ugliest, because they feel that if you touch them, you’ve already infected them”* (+TW17)*.* Only one TW felt that *“stigma has reduced because you can’t see it [HIV infection symptoms]”* (−TW20).

Directly linked to stigma, both MSM and TW repeatedly underlined the fear of rejection as a major barrier when dealing with formal and casual partners. *“He [my boyfriend] told me that his main fear was that, at the moment he would tell me, that I would ghost him”* (−MSM4). Another participant explained that the fear of rejection can lead to hiding the diagnosis: *“He [my boyfriend] told me: ‘I was an asshole for not telling you [my HIV+ status] from the beginning… perhaps it was like part of my denial… I was also really scared of how you would react’”* (−MSM7).

Most TW even added they do not merely fear rejection, but their partner’s violent reactions. *“I would kind of have that fear of, of them against me, you know? … that if they turned out to be positive, they would attack me or do something”* (+TW23). As confirmed by a health care provider: *“their reaction is violent [TW’s male partners’ reaction to notification]”* (C14). TW even spoke of murder as a reaction. *“[A partner] may want to kill me”* (+TW16); *“many people have been killed [for notifying]” (+*TW17); and *“[he] wanted to kill her”* (−TW22). As opposed to TW, only a couple MSM mentioned violent reactions from partners: *“you don’t know if he’ll react in an aggressive way”* (−MSM12).

Another reaction that several TW and a few MSM participants were worried about is having their HIV+ status publicly exposed. *“They will make it public for others, no? ‘This person, don’t get involved with her; she has AIDS now; watch out”* (−TW20). An MSM and a TW also pointed out that the fear of discovering or being accused of an infidelity could be an obstacle.

TW in general described more reactions from partners that could interfere with sharing the diagnosis. One mentioned the fear of being blamed: *“you infected me”* (+TW16); another expressed *“sex becomes something a lot more complicated”* (−TW23). Other emotions considered as obstacles were shame and *“guilt that maybe… I infected my partner”* (−TW23).

MSM and TW frequently identified disinformation as an important barrier or a reason to fear the partner’s reaction: unawareness is often the cause of HIV-related stigma and may, thereby, lead to misunderstandings and rejection.

*I told him and his reaction was: ‘Why you, why you?’ and he started crying … and he was like: ‘No, it’s just that I don’t understand how this can happen to a person like you’… ‘I just don’t know anything about this subject [HIV]; it scares me a lot; and the truth is I, I’d rather, well, not get involved.* (+MSM9)

Knowing more about HIV transmission and its treatment continuum was thought as essential for increasing the acceptance of an HIV diagnosis. Participants argued it provides tools to the person notifying or disclosing, for better communicating the message; and to the partner, to better understand its realistic implications without incurring in fears and negative reactions.

*I would’ve liked him to tell me in person and to tell me based on information, no? like: ‘Ok, look, I just found out I have HIV. You have to get tested… but the treatment is free; nothing will happen to you. If you have HIV, you won’t die… if you get undetectable, you don’t transmit the virus anymore; so the thing of ending up alone won’t happen either’.* (+MSM9)

#### Partner type

Most TW and some MSM thought that having a formal partner is a relevant facilitator for notification or disclosure. TW considered it necessary to inform formal partners, because they share their lives together, and trust and take care of each other. “*The partner that is already with her, the formal partner, I think would understand her, and support her in some way”* (+TW25). MSM thought the beginning of a relationship helps; and that it is easier to inform a formal partner, because with them they have the *“sufficient foundation as to endure the problem”* (−MSM4). Regardless of the partner type, closeness and trust were pointed out by MSM and TW as helpful to inform partners.

At the same time, both MSM and TW identified having casual partners as an obstacle to notification. Both an MSM and a TW agreed they would not know which partners to notify because of the high number of partners and the uncertain time of exposure. *“You don’t know when you caught it [HIV infection]; I mean, how many [casual partners] from back then to inform”* (−MSM12). Participants also explained they often do not have these partners’ contact information, so they are not able to notify them. *“I don’t have a sexual partner that I see often... So no, no, because I don’t even have their information or how to contact them… I wouldn’t do it [notify them]”* (−MSM2). TW mentioned there is no need to disclose to occasional partners, especially in the case of sex work, for they might be put off from sex and there is no emotional attachment to them. *“With my informal partners I don’t have a reason why [to disclose an HIV diagnosis] … I mean, it was only a sexual contact; I would never, never involve feelings at all with someone who is paying me”* (−TW24). However, one TW thought casual partners were easier to inform since those who engage in informal sexual encounters know it is inherently linked with elevated risk. *“I think the informal partner maybe… knows it’s informal, no? And so, maybe, these people already take care of themselves, or are like cautious, as opposed to formal partners. So casual partners maybe already assume a certain risk”* (−TW23).

A couple of MSM believed social networks and dating apps may help find ex-partners they cannot contact anymore: *“if I see them again on Grindr, or on Instagram, or something like that, which is where we have contact, I could do it… send them a message like ‘Hey, this happened and this is my status”* (−MSM2). Dating apps may aid notification, for they have *“a section where they [MSM] can put it [their HIV+ status], and they put it”* (−MSM4). Getting tested regularly and keeping a record of the dates and results was also considered useful by one MSM, because then the person can have a better idea of whom else is at risk and notify them. *“If you keep count… and you’ve been getting tested, and they come out negative, and afterwards one comes out positive, then there you have the range now… then the person that infected you is within that parameter”* (+MSM11). An additional facilitator brought up by an MSM was youth: *“the younger generations… assimilate it better [the HIV diagnosis]*” (−MSM7).

#### Notification/disclosure need

A couple of MSM felt there was no HIV transmission risk and therefore no need to share their diagnosis, due to them not looking sick, being on ART, and always using condoms. *“I had taken perfectly good care of myself with them [used condoms], so I didn’t have a reason to let them know”* (+MSM9). Instead, a TW explained that being on ART may be a facilitator, since *“once on a treatment, she can tell you: ‘Guess what? I do have that disease, but I’m also controlling this disease”* (+TW17). She additionally stated the partner must be informed when there was a risk of HIV transmission, adding that failing to do so may be punished by the law.

*If there is a risk from a condom breaking? Yes [it is good to inform them]. Why? Because now I’m informing him there was a risk, and that risk, although it’s at 4% being undetectable, but it exists. Then you have to inform, so you don’t fall into the crime of hurting the health of another person; and you may even go to jail.* (+TW17)

Other barriers reported by TW included an apparent indifference to transmit HIV: *“they say: ‘oh, I’m already infected, well now let them all get infected, I don’t care’; and they don’t protect themselves” (−TW20);* and vulnerability conditions such as addictions and homelessness. “O*nly a few of them [TW]… have said: ‘My partner told me’… because most are girls [who] lived alcoholism, drug addiction, staying on the streets... and they say: “but I don’t even know who infected me”* (+TW17). This apparent indifference is likely driven by (internalized) stigma and discrimination they face; similarly, substance use and unstable housing are very often the result of discrimination and oppression. Due to these hard circumstances TW might not even know who to notify or who to disclose to.

While most participants mentioned several obstacles for APN, all of them pointed out its benefits and importance. They agreed that APNS could help people accept their diagnosis and overcome barriers for sharing one’s diagnosis, helping them take better care of themselves and their partners. *“If they see a barrier or difficulty [to notify] … to know there’s someone that helps us; I think it is very good; … and maybe that will promote letting partners know, and that is prevention*” (−TW23).

### Suggestions for APNS

Participants stressed that in order for APNS to work properly, more awareness regarding HIV prevention services and HIV in general is needed. One MSM even suggested HIV prevention campaigns for the general public. With regard to APNS, an online communication strategy was proposed to draw people’s attention to the service. Both MSM and TW also suggested that, before the notification moment, the partners could be invited to receive an HIV information and awareness talk, in order to invite them to get tested or to prepare them for their partner’s notification.

*Before notifying them [partners] … first a talk for him: ‘Look, you know this is HIV … it is controlled with this’… Now once he had that talk [informing him about HIV]: ‘You know what? Let’s go to your partner…she was just diagnosed positive with HIV’.* (+TW13)

An MSM indicated the best moment to offer APNS is *“when it [HIV] is diagnosed”* (−MSM2). A TW felt the notification had to happen in a public health institution, for security reasons.

*It would be better in the hospital because… if that partner gets upset, well, there is a precedent too of who her partner was. And if something were to happen to her afterward, we know where to catch [him] or who he was.* (+TW13)

As for the ideal providers for this service, participants suggested counselors and peers—TW also mentioned “*professionals*”, or a multidisciplinary team. *“It would be very interesting if [in] most cases, it was between peers, no? … Peers know what the situation is and how to approach that situation”* (−TW26).

*[About APNS] A psychologist, the medical provider, and a [peer] counselor; sure… Ideally it would be at the same time, no? Like an interview sort of for informing, but at the same time for raising awareness, and at the same time for not stigmatizing… The medical part in which they inform there is nothing to fear if you take care of yourself, if your self-esteem is super cool; and that involves now the psychological part, and the part of, well, supporting, no? And at the end, well, a third party that tells you: ‘Look, I live like this [with HIV], it’s alright, I’m ok, and so can you’.* (−TW24)

Some MSM and most TW said it would be important to have a health care provider that keeps them company during the whole notification process: *“mainly moral support for both persons”* (+MSM1). However, a couple of MSM, who did not have experience being notified, indicated it would be unnecessary to have a health care provider accompany them during notification. *“The other person [being notified] could feel cornered… two people are summoning you to tell you you’re probably sick… it can be taken as somehow a little more aggressive” (*−MSM8*).* Although most TW thought this service does not have to be differentiated between MSM and TW, they stressed that the presence of a provider is necessary to provide a safe environment for avoiding the danger from partner’s violent reactions. *“The most ideal… is to take your partner to Condesa [Mexican HIV clinic] … Because there will be someone that informs them what the disease is about; and, besides, help take care of her personal security”* (+TW15).

Other MSM and TW instead suggested to provide the partner’s contact information so a provider invites them to get tested, through a phone call or finding them in person, without revealing the client’s identity.

*Maybe if one provided information about that person… and they [the providers] came to look for the person and say: … ‘We come to apply tests.’ It would be something easy too, and you protect one [the user, by not revealing the identity] … and the other [the partner] that was diagnosed now.* (+TW13)

One MSM pointed out the usefulness of printed materials with recommendations on how to notify. *“Maybe there could exist like a brochure… of ‘Five steps to tell you are HIV+ to people you don’t know’… and then you decide if you tell or not”* (−MSM12). In addition, the need to work on skills such as communication abilities, including speaking directly and calmly; and personality strengths, such as empathy, self-confidence, and emotional intelligence were mentioned: *“since I’m very self-confident… I could say it [the HIV diagnosis] directly without any problem… It’s like I can manage my emotions a little more… when I’m speaking to someone, I’m like calm”* (−MSM2).

Finally, counselors mentioned it would be important to start training staff for the risks that could arise during APNS. *“You need to have a staff who is fully trained to face the risks of assisted notification in different populations.”* (C18). They also mentioned the importance of raising awareness and empowering the population for the benefits of APNS; by offering support without prejudice and through peers, especially for TW. *“The trans image has a lot of influence for transwomen to access not just the* [HIV] *test but other services.”* (C14) One of them mentioned that civil society organizations could be the right ones to provide APNS. *“*[They] *could have a greater advantage... maybe users can see it as with more ... warmth”* (C27).

### Strategies for implementing APNS

Brainstorming sessions were held with research team members and health professionals with experience in counseling in order to outline strategies that promote the feasibility of implementing APNS.

Specifically, brainstorming sessions identified that although the hypothetical approach for implementing assisted notification services was well accepted, stigma and discrimination, as well as fear of violence associated with the notification process, are major barriers. Therefore, it was deemed important to continue strengthening actions towards mitigating stigma and discrimination associated with HIV. Similarly, HIV screening tests need to be promoted and health workers need training, both for the promotion of these services and for its execution. Hence, a three-step approach was outlined in order to achieve services that meet the needs and context of the study population:

- Strengthening actions to reduce HIV stigma and discrimination: Respondents reported that knowledge about HIV (transmission, importance of testing and treatment, U=U, etc.) may facilitate notification to partners. Therefore, media campaigns were proposed, aiming to demystify HIV infection through evidence-based information, using a TW-inclusive perspective (as opposed to campaigns generally focusing on MSM only).
- Promoting HIV screening tests: Further efforts are needed to promote HIV testing, as fear of the result persists and diagnostic testing is often seen as verification tool rather than prevention. Group discussions among key populations are proposed to educate and raise awareness on the importance of early diagnosis and treatment. Another key component identified was to continue training for health professionals, focusing on all the implications of a diagnosis of HIV infection in order to ensure inclusive pre-counseling.
- Promoting and offering APNS: APNS need to be promoted because they are not well known, even among health professionals. Workshops can be organized with them during which hypothetical notification scenarios are presented and evaluated. However, a distinction should be made between the various levels of need for assistance: while some participants indicated that they would prefer a health professional sharing their status with their partner, others just wanted them to be present to support them during the discussion with their partner. A third group of participants only requested some guiding (e.g., brochures) on how to deliver such a message, but they did not expect a health worker to be present while notifying their status to partners. Furthermore, it is also important to acknowledge that a client might want to give a message related with HIV only, while another client wants to include messages aiming at diminishing stigma or even angry, violent reactions. As such, the profile of the person responsible for providing the service—whether he or she is a medical doctor, psychologist/counselor, or a peer—might differ among clients and needs to be well identified. It might also be useful to include instruments that help determine the risk to which the person notifying might be exposed and to take the required safety measures. An overview of the most important quotes is provided in Annex 2.

## Discussion

Most MSM and TW had not heard about APNS before and they are not a standard practice in Mexico. Regardless, many participants had experience with discussing HIV status with their partners, which was often opportunistic; i.e., triggered by a certain situation, like the start of a relationship or HIV/AIDS-related hospitalizations. Informing only new partners could potentially limit notification or disclosure to formal partners while sharing your HIV status at an advanced stage of the disease could make it difficult to inform partners from the past. In both situations, not all partners are notified in time, especially casual partners. Since APNS should be offered standardly after the diagnosis of HIV, rather than be triggered by external situations, they can improve the timely notification of both formal and casual partners. This could in turn lead to timely HIV testing and diagnosis which is much needed in Mexico; where, in 2018, 11,000 new HIV cases occurred, late linkage to care was calculated at 40% [34], and the HIV mortality rate amounted to four deaths per 100,000 population [20]. In addition, given that notification is already happening, although in an informal manner, it is important to offer tools to ensure that it happens in a safe and trustworthy environment and that the information given is reliable and not discriminatory. APNS could fill in this gap and not only lead to more notifications but also to better informed partners.

The main barrier for partner notification and serostatus disclosure was the fear of negative consequences associated with HIV-related stigma. A study in Australia similarly reported that, because of prevailing HIV stigma, most MSM were very selective about who they disclosed their status to, often only telling partners perceived at risk [35]. Nevertheless, since most participants in our study had experiences with informal partner notification despite stigma concerns, Mexican health care institutions could use this willingness to share an HIV diagnosis with partners and provide professional services that help avoid or mitigate stigma’s consequences. As such, more partners might be notified in time. However, services do need to be adapted to the needs of the target groups. This was also concluded by a study among black or Latino MSM and TW that used APNS in North Carolina (USA): they worried about stigma and privacy, and generally perceived the service as aggressive; but empathy, choice, autonomy, and support with navigating services led to positive experiences [36]. Accordingly, education and awareness campaigns to eliminate HIV-related stigma are an important first step to prepare the ground for APNS. Nevertheless, APNS may in itself also contribute to reduce stigma by providing notified partners an adequate counseling.

Another important aspect to highlight was the concern about partner’s violent reactions, which was often a significant barrier for notification and serostatus disclosure, especially among TW. Several studies have confirmed that LGBT people (trans in particular) tend to experience more verbal or physical partner violence, compared to heterosexuals and cisgender people [37, 38]. Estimating PLWH’s vulnerability will be important to ensure that APNS can mitigate aggressive or harmful reactions.

Stigma and other barriers for notification represent challenges for the implementation of APNS, but notification facilitators could leverage the difficulties. One important facilitator is the positive perception regarding APNS, because, notwithstanding the barriers, our participants believed this service would have benefits and often expressed they are important and acceptable. Nevertheless, a study that implemented a partner notification model among MSM and TW living with HIV in Tijuana, Mexico—made available after our data was collected—reported a lower-than-expected partner notification uptake, due to problems identifying HIV care clients willing to participate, obtaining reliable partner contact information, and engaging notified partners. Still, they recruited 36 clients who listed 115 sexual partners for either provider (i.e., providers notify partners; 94%) or client referral (i.e., clients notify partners; 6%); 70% of those listed were notified and 76% of those notified agreed to be screened [39]. For increasing the acceptance of APNS and subsequent notification of partners, one strategy that significantly worked among MSM in Australia was the use of opt-out referral (i.e., all users diagnosed with HIV are referred to APNS unless they decline it) instead of opt-in referral (i.e., users are offered the referral) [40]. However, the use of the opt-out referral in Mexico would have to be evaluated given the context of stigma and discrimination against HIV key populations. As for overcoming the difficulty of contacting casual partners, one option recommended by other studies—and suggested by MSM in our findings as well—is geosocial networking app-based notification, which MSM have shown high willingness to use, particularly with an anonymous setting [41–43]. Finally, a last consideration for the implementation of APNS in Mexico is that most of the states’ criminal codes contemplate putting others at risk for infection as a crime [44]; APNS should be implemented in all HIV/AIDS services to help avoiding potential legal cases.

There are some limitations to this study. First, we focused on MSM and TW in Mexico City, so factors pertaining partner notification may differ in other high-risk groups and geographic locations. Second, by including both MSM and TW we couldn’t verify in-depth the needs of both groups. Our research did point out that TW were more afraid of violent reactions; given that MSM and TW can differ socially it would be recommended to focus on them separately. Third, selecting participants at ASOs means we did not reach those without the willingness to attend such services, who could have different views on APNS. We nonetheless reached saturation in the explored topics and believe these results are transferrable to similar contexts. Lastly, given the absence of APNS in Mexico, the explored experiences and factors reflected informal partner notification and serostatus disclosure, not assisted partner notification directly. If APNS were to be implemented in Mexico, further studies can expand on these findings by assessing direct experiences with this service.

## Conclusions

Implementing APNS for MSM and TW in Mexico is feasible if properly designed. Despite the concerns informants voiced regarding sharing one’s HIV status, all participants saw APNS as beneficial, which could increase their acceptance likelihood if implemented. However, when designing APNS, strategies may need to be flexible, allowing for a more personalized approach, and addressing differential needs and vulnerabilities. Also, professionally executed services are necessary to help mitigate obstacles like lack of awareness regarding HIV, fear of rejection or even violent reactions. Finally, APNS should be implemented along with other strategies aimed to increase HIV knowledge, and to reduce stigma and discrimination.

## Supplementary Information

**Additional file 1.** The interview guides developed for this study can be found as an additional file.

**Additional file 2.** The document contains the interview guides for the interview with the MSM and TW, as well as the guides for the counselors. Also an overview of the most important quotes is provided.

## Data Availability

The datasets generated and/or analyzed during the current study are not publicly available due to confidentiality concerns, since the staff and participants of ASOs in Mexico City come from a minority and could potentially be identified through the full contents of the interview transcripts. However, the datasets are available from the corresponding author on reasonable request.

## Abbreviations

AIDS: Acquired immunodeficiency syndrome
APNS: Assisted partner notification services
ART: Antiretroviral therapy
ASO: AIDS Service Organization
C: Counselors
HIV: Human immunodeficiency virus
LGBT: Lesbian, gay, bisexual, and transgender
MSM: Men who have sex with men
PLWH: People living with HIV
TW: Transwomen
UNAIDS: Joint United Nations Program on HIV/AIDS
