# Supplementary material for: Developing HIV assisted partner notification services tailored to Mexican key populations: a qualitative approach
Source: BMC Public Health. 2021 Mar 20;21:555. doi: 10.1186/s12889-021-10612-3 (PMC7981994; doi:10.1186/s12889-021-10612-3)
Supplement: Supplementary file 1 — Additional file 1. The interview guides developed for this study can be found as an additional file. [file 12889_2021_10612_MOESM1_ESM.docx]

**Developing HIV Assisted Partner Notification Services Tailored to Mexican Key Populations: A Qualitative Approach**

## Interview Guide for MSM/TW

**General information of the interview (only to be mentioned on the recording):**

● Interview date

● Interview number

**●** Clinic

**Introduction**

My name is _____. I am from the National Institute of Public Health and I belong to a research team that is studying what elements need to be included in an intervention for the notification of partners of people that were diagnosed with HIV. We are very interested in listening to your ideas, perceptions, and suggestions about this.

As I explained previously, this conversation is completely anonymous and confidential. What you and I talk about here will not be reported to other people that are not participating directly in the project and there will be no way to associate this information with your personal data. It is important to remind you that you have the right to not answer any question you do not want to, and that would not be a problem. The interview will take approximately 30-40 minutes.

Since we need to analyze the information that we speak about today, we need to record the interview. This recording will be stored under a password and only the research team will have access to it. When we analyze the information, we will use a code instead of your name. Do you agree to having this interview recorded?

The consent form that you signed contains the information of those responsible for the study and of the Ethical Committee from the National Institute of Public Health, in case you have any doubts after the interview. Up to this moment, do you have and questions?

**Experience with HIV Partner Notification and its Barriers/Facilitators**

First, I would like to ask you some questions about any experience you have had with HIV partner notification, including what has made this experience harder or easier.

HIV+ participants:

1. When you were diagnosed with HIV, did someone recommend that you let any of your partners know about your HIV status?

1. *If yes:* What were you told? What happened at that time? How did you feel? What do you think of that experience? Would you have changed something about that process?

2. *In case they were not recommended or offered the notification service for their partners:*

1. Do you think it would be good to let know or notify people in case that any of their partners had an HIV-positive test? *Delve into the reasons.*
2. Would you have liked someone to recommend you to do it when you were diagnosed with HIV? *Delve into the reasons.*
3. Would you have liked someone to help you notify your partners about your HIV diagnosis? *Delve into the reasons.*
4. By which means would you have liked your partner(s) to be contacted so they can be notified? *(E.g., phone, email, text message, in person, etc.)* *or* By which means do you think partners should be contacted? *Delve into the reasons.*

3. Since you were diagnosed, have you let know or notified any of your partners that you are living with HIV?

1. *If not:* Have you thought about letting them know at some point? Which sex partners have you thought about notifying? How do you think you could notify them? What would motivate you or discourage you to do so? What things cross your mind when thinking about the possibility of letting them know?
2. *If yes:* Could you tell me how you let them know? Was it in person or through other means? How did your partner react? What was the most difficult thing? Was there something that made it easier to tell them? Was there something unexpected when notifying your partners? Is there something you would have changed or would have liked different at that time? Would you have liked to have someone help or support you?

HIV- participants:

4. When you were tested for HIV or other STIs, if you had received a positive result, would you have let your partners know? How would you do it? Would you ask for help? To whom?

All participants:

5. Have you been notified by a partner that he/she was living with HIV?

1. *If yes:* How was it? How did you feel? Would you have changed anything?
2. *If not:* Would you have liked any of your partners to notify you that they were living with HIV? How would you have liked to be told? Where? By which means (in person, at a distance)? By whom would you have liked to be notified?

6. Do you know somebody who was notified by a partner that they were living with HIV? What did you hear about it? What do you think about it?

7. Let’s imagine you have a friend called Erick/Lucía, who just found out he/she is living with HIV and is thinking about letting his/her recent sex partners know about it. Erick/Lucía reaches out to you and asks for your opinion regarding doing this or not.

1. What would you tell him/her?
2. Who do you think he/she should notify? *Delve into the reasons.*
3. What barriers do you think that Erick/Lucía may find in doing so?
4. In which way do you think Erick/Lucía can notify their partners?
5. What things do you think would make the process easier for Erick/Lucía to notify their partners?

**Suggestions for Assisted Partner Notification Services**

To end this interview, I would like to ask you a few questions regarding the possibility of implementing a service for the notification of people living with HIV’s partners.

8. Whom would you prefer to assist people recently diagnosed with HIV during the notifications service? Medical providers, the provider who applied the HIV test, or perhaps other HIV patients with experience in the matter? *Delve into the reasons.*

9. At which point would you prefer to be offered the partner notifications service? At the time of the HIV diagnosis, during the following clinic visit, or perhaps once you have started taking the HIV treatment? *Delve into the reasons.*

10. What benefits do you think that the partner notification service could have? What disadvantages could it have? *Delve into the reasons.*

We have reached the end of the interview. Is there anything else you would like to add?

*Thank the participant for the interview and end the recording.*

## Interview Guide for health care provider

**General information of the interview (only to be mentioned on the recording):**

● Interview date

● Interview number

**●** Clinic

**Introduction**

My name is _____. I am from the National Institute of Public Health and I belong to a research team that is studying what elements need to be included in an intervention for the notification of partners of people that were diagnosed with HIV. We are very interested in listening to your ideas, perceptions, and suggestions about this.

As I explained previously, this conversation is completely anonymous and confidential. What you and I talk about here will not be reported to other people that are not participating directly in the project and there will be no way to associate this information with your personal data. It is important to remind you that you have the right to not answer any question you do not want to, and that would not be a problem. The interview will take approximately 30 minutes.

Since we need to analyze the information that we speak about today, we need to record the interview. This recording will be stored under a password and only the research team will have access to it. When we analyze the information, we will use a code instead of your name. Do you agree to having this interview recorded?

The consent form that you signed contains the information of those responsible for the study and of the Ethical Committee from the National Institute of Public Health, in case you have any doubts after the interview. Up to this moment, do you have and questions?

**Experience with HIV Partner Notification and its Barriers/Facilitators**

I would like to ask you some questions about any experience you have had with HIV partner notification, including what has made this experience harder or easier.

1. First, can you please describe for me the process for HIV testing and counselling at the clinic/organization you work at? Starting from the person’s arrival and until they get an HIV diagnosis and leave. *Delve into each step.*

2. During this process, for those who get diagnosed with HIV, do you recommend them to let their partners know about their diagnosis? *Explore the frequency of this and possible barriers. Ask them to provide an example of how they recommend partner notification.*

3. What has been the reaction of patients when you recommended this? What have you been told usually? What do you think about that?

4. And now regarding the clinic/organization where you work, do patients get offered support for letting their partners know about their HIV diagnosis? *Delve into the logistic and frequency of this. Ask if they can provide an example of how this process is carried out.*

1. What is the reaction of patients when they get offered this service?
2. What do they tell patients usually?
3. What do HIV+ patients think about that?

5. Had you previously heard about the HIV partner notification service? What have you heard about it?

6. Have you received training or talks about how to recommend partner notification to people or about how to notify the partners of people diagnosed with HIV? How did it go? What did it include?

7. Have you had to notify the partner of a patient about their HIV diagnosis? How was it? What steps did you follow to do so? Were there any difficulties? What was the reaction of the partner?

8. What do you think about assisted partner notification (for or against it)?

**Suggestions for Assisted Partner Notification Services**

To end this interview, I would like to ask you a few questions regarding the possibility of implementing a service for the notification of people living with HIV’s partners.

1. What things should be taken into account before implementing such a service?

2. Do you think it would work? *Delve into the reasons.*

1. *If not:* What would you suggest for it to work?

3. What barriers do you think would be found when trying to carry out this service?

4. According to your experience in the matter, who do you think should assist people that have been recently diagnosed with HIV during the notification service? *Delve into possible profiles, e.g., medical providers, testing providers, or other HIV patients*.

5. When do you think would be the most appropriate moment to offer the partner notification service? At the time of the HIV diagnosis, during the following clinic visit, or once HIV treatment is started? *Delve into the reasons.*

11. What benefits do you think that the partner notification service could have? What disadvantages could it have? *Delve into the reasons.*

We have reached the end of the interview. Is there anything else you would like to add?

*Thank the participant for the interview and end the recording.*
