# Supplementary material for: Developing HIV assisted partner notification services tailored to Mexican key populations: a qualitative approach
Source: BMC Public Health. 2021 Mar 20;21:555. doi: 10.1186/s12889-021-10612-3 (PMC7981994; doi:10.1186/s12889-021-10612-3)
Supplement: Supplementary file 2 — Additional file 2. The document contains the interview guides for the interview with the MSM and TW, as well as the guides for the counselors. Also an overview of the most important quotes is provided. [file 12889_2021_10612_MOESM2_ESM.docx]

**Developing HIV Assisted Partner Notification Services Tailored to Mexican Key Populations: A Qualitative Approach**

**Table . Sample of quotes of barriers and facilitators for HIV partner notification and serostatus disclosure among MSM and TW**

| ***Themes*** | ***Barriers*** | ***Facilitators*** |
| --- | --- | --- |
| *Partner reactions* | - **Fear of negative reactions** (stigma, rejection, violence, and exposure):  *“Unfortunately, there’s people that are still living with the taboo; and the taboo I think is the ugliest, because they feel that if you touch them, you’ve already infected them”* (+TW17)*.*  **- Disinformation:**  *“I told him and his reaction was: ‘Why you, why you?’ and he started crying … and he was like: ‘No, it’s just that I don’t understand how this can happen to a person like you’… ‘I just don’t know anything about this subject [HIV]; it scares me a lot; and the truth is I, I’d rather, well, not get involved”* (+MSM9).  **- Negative emotions:**  *“Guilt that maybe… I infected my partner”* (-TW23). | - **Information:**  *“I would’ve liked him to tell me in person and to tell me based on information, no? like: ’Ok, look, I just found out I have HIV. You have to get tested… but the treatment is free; nothing will happen to you. If you have HIV, you won’t die… if you get undetectable, you don’t transmit the virus anymore; so the thing of ending up alone won’t happen either’”* (+MSM9). |
| *Partner type* | **- Casual partner:**  *“You don’t know when you caught it [HIV infection]; I mean, how many [casual partners] from back then to inform”* (-MSM12).  **- Missing partner’s contact information:**  *"I don't have a sexual partner that I see often... So no, no, because I don't even have their information or how to contact them… I wouldn't do it [notify them]"* (-MSM2).  **- Lack of emotional attachment:**  *“With my informal partners I don’t have a reason why [to disclose an HIV diagnosis] … I mean, it was only a sexual contact; I would never, never involve feelings at all with someone who is paying me”* (-TW24). | **- Formal partner:**  “*The partner that is already with her, the formal partner, I think would understand her, and support her in some way”* (+TW25).  **- Casual partner:**  *"I think the informal partner maybe… knows it’s informal, no? And so, maybe, these people already take care of themselves, or are like cautious, as opposed to formal partners. So casual partners maybe already assume a certain risk”* (-TW23).  **- Apps/social networks:**  *“A section where they [MSM] can put it [their HIV+ status], and they put it”* (-MSM4).  **- Regular testing:**  *“If you keep count… and you’ve been getting tested, and they come out negative, and afterwards one comes out positive, then there you have the range now… then the person that infected you is within that parameter”* (+MSM11).  **- Youth:**  *“The younger generations… assimilate it better [the HIV diagnosis]*” (-MSM7). |
| *Notification /Disclosure need* | **- Non-risk perception (including taking ART):**  *“I had taken perfectly good care of myself with them [used condoms], so I didn’t have a reason to let them know”* (+MSM9).  - **Apparent indifference:**  *“They say: ‘oh, I’m already infected, well now let them all get infected, I don’t care’; and they don’t protect themselves” (-TW20).*  **- Vulnerability conditions:**  “O*nly a few of them [TW]… have said: ‘My partner told me’… because most are girls [who] lived alcoholism, drug addiction, staying on the streets... and they say: “but I don’t even know who infected me* (+TW17).” | **- Risk perception:**  *“If there is a risk from a condom breaking? Yes [it is good to inform them]. Why? Because now I’m informing him there was a risk, and that risk, although it’s at 4% being undetectable, but it exists”* (+TW17)  **- Taking ART:**  *“Once on a treatment, she can tell you: ‘Guess what? I do have that disease, but I’m also controlling this disease”* (+TW17).  **- Benefits perception:**  *“If they see a barrier or difficulty [to notify] … to know there’s someone that helps us; I think it is very good; … and maybe that will promote letting partners know, and that is prevention*” (-TW23). |
| *APNS strategies* | **- Support from a provider:**  *"The other person [being notified] could feel cornered… two people are summoning you to tell you you’re probably sick… it can be taken as somehow a little more aggressive" (*-MSM8*).*  **- Maintaining the client’s anonymity:**  *“Maybe if one provided information about that person… and they [the providers] came to look for the person and say: … ‘We come to apply tests.’ It would be something easy too, and you protect one [the user, by not revealing the identity] … and the other [the partner] that was diagnosed now”* (+TW13).  **- Printed materials:**  *“Maybe there could exist like a brochure… of ‘Five steps to tell you are HIV+ to people you don’t know’… and then you decide if you tell or not”* (-MSM12).  **- Promoting communication skills:**  *“Since I’m very self-confident… I could say it [the HIV diagnosis] directly without any problem… It’s like I can manage my emotions a little more… when I’m speaking to someone, I’m like calm”* (-MSM2). | ***- Handling stigma and disinformation***  *“Before notifying them [partners] … first a talk for him: ‘Look, you know this is HIV … it is controlled with this’… Now once he had that talk [informing him about HIV]: ‘You know what? Let’s go to your partner…she was just diagnosed positive with HIV’”* (+TW13).  ***- A safe location:***  *“It would be better in the hospital because… if that partner gets upset, well, there is a precedent too of who her partner was. And if something were to happen to her afterward, we know where to catch [him] or who he was”* (+TW13).  **- Support from a provider or counsellor:**  *“A psychologist, the medical provider, and a [peer] counselor”* (-TW24). |
